# Protection against Doxorubicin-Related Cardiotoxicity by Jaceosidin Involves the Sirt1 Signaling Pathway

**DOI:** 10.1155/2021/9984330

**Published:** 2021-08-06

**Authors:** Yuzhou Liu, Liying Zhou, Binbin Du, Yuan Liu, Junhui Xing, Sen Guo, Ling Li, Hongrui Chen

**Affiliations:** Department of Cardiology, The First Affiliated Hospital of Zhengzhou University, Zhengzhou, Henan 450052, China

## Abstract

The clinical use of doxorubicin (DOX) is largely limited by its cardiotoxicity. Previous studies have shown that jaceosidin has many biological activities. However, little is known about whether jaceosidin can attenuate DOX-related acute cardiotoxicity. Here, we investigated the therapeutic effects of jaceosidin on DOX-induced acute cardiotoxicity. Mice were intraperitoneally injected with a single dose of DOX to establish an acute cardiac injury model. To explore the protective effects, mice were orally administered jaceosidin daily for 7 days, with dosing beginning 2 days before DOX injection. The results demonstrated that jaceosidin dose-dependently reduced free radical generation, inflammation accumulation, and cell loss induced by DOX in cardiomyocytes. Further studies showed that jaceosidin treatment inhibited myocardial oxidative damage and the inflammatory response and attenuated myocardial apoptotic death, thus improving cardiac function in mice injected with DOX. The inhibitory effects of jaceosidin on DOX-related acute cardiotoxicity were mediated by activation of the sirtuin1 (Sirt1) signaling pathway. Jaceosidin lost its protective effect against DOX-related injury in Sirt1-deficient cardiomyocytes and mice. In conclusion, jaceosidin has protective potential in treating DOX-related cardiac injury through activation of the Sirt1 signaling pathway.

## 1. Introduction

Doxorubicin (DOX) has been widely used to treat solid and haematopoietic tumours; however, a major limiting factor for the clinical use of DOX is irreversible cardiac toxicity. DOX-induced cardiotoxicity is characterized by irreversible degenerative cardiomyopathy and congestive heart failure [1–3]. The pathogenesis of cardiotoxicity induced by DOX remains poorly understood, but accumulating evidence suggests the indispensable roles of free radical production and myocardial apoptosis [4]. DOX-induced cardiomyopathy occurs primarily through the generation of reactive oxygen species (ROS), which can induce myocardial lipid peroxidation and myofibre degeneration [5, 6]. Additionally, previous studies have linked ROS production to cardiomyocyte apoptosis [7]. It has been demonstrated that DOX-induced cardiotoxicity can be suppressed by the overexpression of superoxide dismutase (SOD) and catalase [8, 9]. These findings suggest that ROS production and myocardial apoptosis play important roles in DOX-induced cardiotoxicity. Therefore, we speculated that the suppression of ROS production and apoptotic cell death might largely rescue DOX-triggered cardiotoxicity.

Jaceosidin is a flavone isolated from medicinal plants of the genus *Artemisia* [10]. Jaceosidin has recently gained attention as a beneficial drug with low intrinsic toxicity. Moreover, jaceosidin has been demonstrated to possess antioxidative, anti-inflammatory, and immunosuppressive properties [11–13]. A recent study found that jaceosidin inhibits the inflammatory response and decreases complement levels in lipopolysaccharide- (LPS-) injected mice [14]. Fu et al. found that jaceosidin scavenged ROS and weakened mitochondrial lipid peroxidation in isolated rat livers [15]. In addition, jaceosidin ameliorated endoplasmic reticulum stress and insulin resistance by upregulating sarco-endoplasmic reticulum Ca2+-ATPase 2b [16]. However, the effects of jaceosidin on DOX-related cardiac injury and the related signaling mechanisms remain unclear. Given the roles of inflammation and oxidative damage in DOX-related cardiac injury, the present study investigated whether jaceosidin attenuates DOX-related cardiac injury in vivo and in vitro.

## 2. Methods

### 2.1. Reagents

Jaceosidin (cat. no. 18085-97-7) and DOX (cat. no. 1515) were purchased from Sigma-Aldrich (St. Louis, MO, USA). Antibodies against nuclear factor E2-related factor 2 (Nrf2, cat. no. 12721S; 1 : 1000 dilution), haem oxygenase-1 (HO-1, cat. no. 43966; 1 : 1000 dilution), nuclear factor kappa-B (NF-*κ*B, cat. no. 8242; 1 : 1000 dilution), phospho-I*κ*B kinase *β* (P-IKK*β*; cat. no. 2078; 1 : 1000 dilution), IKK*β* (cat. no. 2687; 1 : 1000 dilution), Bax (cat. no. 5023; 1 : 1000 dilution), Bcl-2 (cat. no. ab32124; 1 : 1000 dilution), sirtuin1 (Sirt1, cat. no. 8469; 1 : 1000 dilution), and GAPDH (cat. no. 5174; 1 : 1000 dilution) were obtained from Cell Signaling Technology Inc. (Danvers, MA, USA).

### 2.2. Cell Culture and Treatment

Primary cultures of neonatal rat cardiac myocytes (NRCMs) were prepared as previously described [17]. The cells were cultured in Dulbecco's modified Eagle's medium supplemented with 10% heat-inactivated foetal bovine serum (FBS; Gibco; Thermo Fisher Scientific, Inc., Waltham, USA). Jaceosidin was dissolved in 0.1% dimethylsulfoxide (DMSO) for use in all in vitro experiments. To induce DOX-related injury, the cells were starved overnight in DMEM containing 0.5% FBS and then incubated with DOX (1 *μ*mol/L) for 24 h. The cells were pretreated with different concentrations of jaceosidin (0, 2.5, 5, 10, 15 *μ*mol/l) 6 hours before DOX administration. The dose of jaceosidin was determined according to a previous study [16]; the dose of DOX was also selected according to a previous study [2]. To knock down Sirt1 in cardiomyocytes, NRCMs were preincubated with siSirt1 (50 nmol/l) or siRNA (50 nmol/l) for 24 h and then subjected to DOX treatment for 24 h. siSirt1 and scrambled siRNA were obtained from Invitrogen. Cell viability was determined by a cell counting kit- (CCK-) 8 kit. Briefly, CCK-8 (10 *μ*l) was added to the medium (100 *μ*l) in each well of a 96-well plate and then incubated at 37°C for 2.5 hours. Optical density values were obtained at 450 nm using a BioTek Synergy HT Multi-Mode Microplate Reader.

### 2.3. Animals and Treatment

A total of 48 male C57BL/6 mice (age: 8-9 weeks, weight: 22-24 g) were purchased from the Experimental Animal Center of Zhengzhou University (Henan, China). The experimental protocols were approved by the Committee on Animal Care of The First Affiliated Hospital of Zhengzhou University (No. 8196437). All mice were allowed free access to food and water and were maintained on a 12 h light/dark cycle in a controlled temperature (20-25°C) and humidity (50 ± 5%) environment. After 1 week of adaptation, the mice were divided into four groups (*n* = 12 each): (1) vehicle+saline group, in which mice were orally administered 0.5% carboxymethylcellulose solution (vehicle) daily for 7 days and received a saline injection on the second day of vehicle administration; (2) jaceosidin+saline group, in which mice were orally administered jaceosidin (4 mg/kg) daily for 7 days and received a saline injection on the second day of jaceosidin administration; (3) vehicle+DOX group, in which mice were orally administered 0.5% carboxymethylcellulose solution (vehicle) daily for 7 days and intraperitoneally injected with a single dose of DOX (15 mg/kg) on the second day of vehicle administration; and (4) jaceosidin+DOX group, in which mice were orally administered jaceosidin (4 mg/kg) daily for 7 days and intraperitoneally injected with a single dose of DOX (15 mg/kg) on the second day of jaceosidin administration. The dose of DOX was determined according to a previous study [2]. The jaceosidin suspension was prepared in 0.5% carboxymethylcellulose for the animal experiments. The dose of jaceosidin was selected according to a previous study [16]. Five days after DOX injection, the mice were sacrificed with a single overdose of pentobarbital sodium (200 mg/kg, ip), and blood samples and heart tissues were collected for further experiments.

To inhibit Sirt1 in vivo, mice were administered a specific inhibitor of Sirt1 (Ex527, 1 mg/kg) every other day for a total of 8 days beginning 3 days before DOX injection. The dose of Sirt1 was selected according to a previous study [18].

### 2.4. Haemodynamics Analysis

Cardiac function was assessed by a pressure-volume catheter at 5 days after DOX injection. Mice (*n* = 8 per group) were anaesthetized with ketamine (100 mg/kg) and xylazine (2.5 mg/kg) and then connected to a rodent ventilator after endotracheal intubation [19]. Cardiac catheterization was then performed using a 1.0-F catheter (SPR 839; Millar Instruments Inc.) inserted retrograde through the right carotid artery into the left ventricle. After stabilization for 10 min, the pressure signals and heart rate were recorded continuously with an ARIA pressure-volume conductance system coupled to a Powerlab/4SP A/D converter. The data were analysed using the LabChart software. All experiments were carried out in a blinded manner, and the data were analysed as previously described.

### 2.5. Western Immunoblot

Proteins from the heart samples were extracted with RIPA buffer. Nuclear proteins were extracted with NE-PER™ Nuclear and Cytoplasmic Extraction Reagents (Invitrogen). Proteins were separated by 10% SDS-PAGE and transferred onto polyvinylidene fluoride membranes. After blocking with 5% skim milk for 4 h at room temperature, the membranes were incubated with the primary antibodies at 4°C overnight. After incubation with the secondary antibody (1 : 10000, Thermo Fisher Scientific) for 2.5 hours at room temperature, the protein bands on the membranes were scanned using an enhanced chemiluminescence system and a BioSpectrum gel imaging system (CA, USA). Secondary antibodies and the enhanced chemiluminescence solution were purchased from Amersham. The band intensity was quantified by the ImageJ software, and GAPDH was used as the internal reference.

### 2.6. Real-Time Polymerase Chain Reaction

Total RNA was extracted using the RNeasy mini kit (Qiagen). cDNA was generated using Superscript III reverse transcriptase and random primers (Invitrogen). Real-time polymerase chain reaction (PCR) was performed using LightCycler 480 SYBR Green Master Mix (Roche Diagnostics). GAPDH was used as the internal reference.

### 2.7. Cell Injury Assay

Five days after DOX injection, blood samples were collected from the retro-orbital plexus of mice. The mouse cTnI ELISA kit (#CSB-E08421m) and mouse LDH ELISA kit (#CSB-E17733m) were purchased from CUSABIO. Plasma cardiac troponin I (cTnI) and lactate dehydrogenase (LDH) were detected using commercial kits according to the manufacturer's instructions. Optical density values were obtained at 450 nm using a BioTek Synergy HT Multi-Mode microplate reader. To evaluate cardiomyocytes in vitro, creatine kinase (CK) was detected using a CK assay kit (A032-1-1, Nanjing Jiancheng Bioengineering Institute).

### 2.8. Oxidative Status and Myocardial Cytokines

Five days after DOX injection, fresh heart samples were collected and homogenized to detect malondialdehyde (MDA) content, 4-hydroxynonenal (4-HNE), and total SOD activity using commercially available kits according to the manufacturer's instructions. Nrf2 binding activity was detected using the Nrf2 DNA binding ELISA kit (Active Motif). Cardiomyocytes were also collected to detect intracellular ROS, hydrogen peroxide, and superoxide production. The ROS assay kit was obtained from Abcam (#ab186027). This kit provides an ultrasensitive fluorometric one-step ROS assay that can be performed in a 96-well microtiter plate format. The signal was read by a fluorescence microplate reader at Ex/Em = 520/605 nm. The hydrogen peroxide assay kit was obtained from Biovision (Shanghai, China). This kit provides a highly sensitive, colorimetric assay for measuring H_₂_O_₂_ in biological samples. In this assay protocol, horseradish peroxidase (HRP) reacts with H_₂_O_₂_ to produce a product with red fluorescence (Ex/Em = 535/587 nm). The superoxide assay kit was obtained from Beyotime (Beijing, China). The protein carbonyl content in the supernatants of heart tissue homogenates was determined by using a protein carbonyl content assay Kit (K830-100, Biovision). The resulting signal was read by a fluorescence microplate reader at 375 nm. Oxidized glutathione (GSSG) and total glutathione concentrations were detected using a total glutathione/oxidized glutathione assay kit (A061-1-1; Nanjing Jiancheng Bioengineering Institute). Reduced glutathione (GSH) values were determined from the total and GSSG concentrations. The redox status was represented by the GSH/GSSG ratio.

The DNA-p65 NF-*κ*B binding assay was performed with a Mercury TransFactor kit (BD Biosciences, Clontech). In addition, fresh heart samples were collected and homogenized to detect myocardial tumour necrosis factor- (TNF-) *α* and interleukin- (IL-) 6 expression. The TNF-*α* mouse ELISA kit (#BMS607-3) and IL-6 mouse ELISA kit (#BMS603HS) were purchased from Invitrogen.

### 2.9. HE Staining, TUNEL Analysis, and Caspase-3 Activity

For histological analysis, hematoxylin and eosin (H&E) staining was used. Fresh heart samples were sectioned to detect myocardial apoptosis with TdT-mediated dUTP nick end-labelling (TUNEL) using a CardioTACS kit (R&D Systems) according to the manufacturer's instructions. Cardiac caspase-3 activity was also measured with a CPP32/caspase-3 colorimetric protease assay.

### 2.10. Serum Transaminases and Creatinine Analyses

To evaluate the potential oral toxicity of jaceosidin, mice were orally administered jaceosidin (4 mg/kg) daily for 7 days. After that, the mice were sacrificed, and blood samples were collected to detect serum transaminases and creatinine. The kit for alanine transaminase (ALT), glutamate pyruvate transaminase (AST), and creatinine were provided by Nanjing Jiancheng Bioengineering Institute (Nanjing, China).

### 2.11. Statistical Analysis

The data are presented as the mean ± SEM. Statistical comparisons between two groups were performed using two-tailed Student's *t*-tests. Comparisons between multiple groups were performed using one-way ANOVA followed by a post hoc Bonferroni comparison analysis. Statistical significance was accepted at a value of *P* < 0.05.

## 3. Results

### 3.1. Jaceosidin Treatment Suppressed Intracellular ROS in DOX-Treated Cardiomyocytes

**Figure 1:**
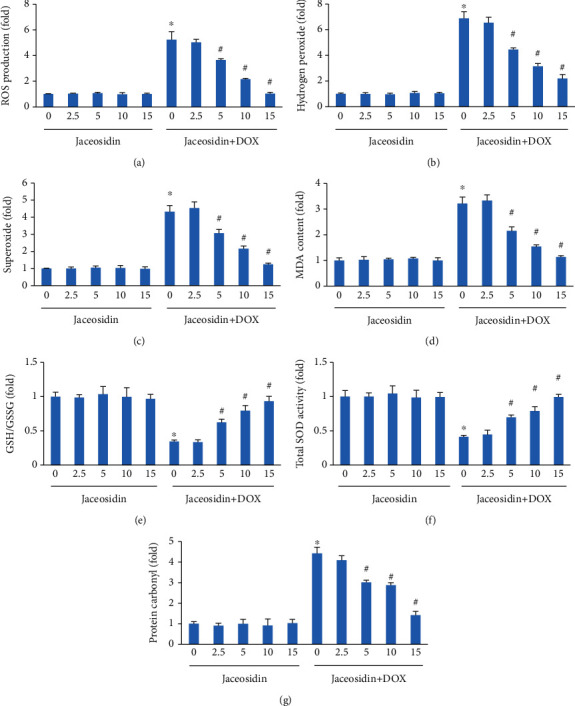
Jaceosidin inhibited reactive oxygen species (ROS) production in doxorubicin- (DOX-) treated cells. (a–c) The production of ROS, hydrogen peroxide, and superoxide in jaceosidin-treated cells (*n* = 6). (d) Malondialdehyde (MDA) content in jaceosidin-treated cells (*n* = 6). (e) The ratio of glutathione (GSH) to oxidized glutathione (GSSG) in jaceosidin-treated cells (*n* = 6). (f) Total superoxide dismutase (SOD) activity in DOX-treated cells. (g) Protein carbonyl content in the indicated groups (*n* = 6). For (a–g), cells were pretreated with various concentrations of jaceosidin (0, 2.5, 5, 10, 15 *μ*mol/l) 6 hours before DOX (1 *μ*mol/L) administration. The oxidative stress markers (a–g) were detected 24 hours after DOX administration. Data are shown as means ± SEM. Comparisons between multiple groups were performed using one-way ANOVA followed by a post hoc Bonferroni comparison analysis. ^∗^*P* < 0.05 compared with control. ^#^*P* < 0.05 compared with DOX alone.

To investigate the effects of jaceosidin, NRCMs were pretreated with jaceosidin at different concentrations and subsequently treated with DOX for 24 hours. Jaceosidin significantly decreased the ROS production induced by DOX in a dose-dependent manner, with a maximal effect at a dose of 15 *μ*mol/l (Figure 1(a)). We further investigated the inhibitory effect of jaceosidin on hydrogen peroxide and superoxide production in DOX-treated cells. We found that the elevations in hydrogen peroxide and superoxide in cardiac myocytes in response to DOX were significantly attenuated after jaceosidin treatment (Figures 1(b) and 1(c)). In addition, jaceosidin treatment markedly reduced myocardial MDA levels in DOX-treated cells (Figure 1(d)). DOX decreased the GSH/GSSG ratio and the total SOD activity; however, these alterations were largely inhibited by jaceosidin in a dose-dependent manner (Figures 1(e) and 1(f)). We also detected the protein carbonyl content, which is a representative product of protein oxidative damage. The results showed that jaceosidin dose-dependently decreased the protein carbonyl content in DOX-treated cells (Figure 1(g)).

### 3.2. Jaceosidin Treatment Suppressed Inflammation and Cell Loss in DOX-Treated Cardiomyocytes

**Figure 2:**
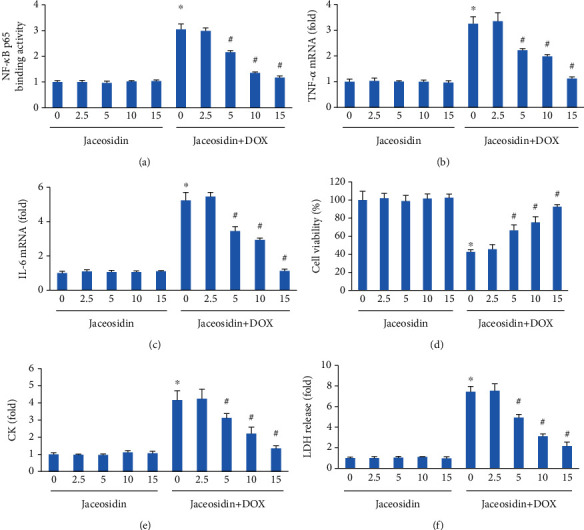
Jaceosidin inhibited the inflammatory response and cell loss in vitro. (a) Nuclear factor kappa-B (NF-*κ*B) binding activity (*n* = 6). (b, c) The mRNA levels of inflammatory factors (*n* = 6). (d) Cell viability after doxorubicin (DOX) treatment (*n* = 6). (e, f) The release of lactate dehydrogenase (LDH) and creatine kinase (CK) in cells (*n* = 6). For (a–f), cells were pretreated with various concentrations of jaceosidin (0, 2.5, 5, 10, 15 *μ*mol/l) 6 hours before DOX (1 *μ*mol/L) administration. The inflammatory markers (a–f) were detected 24 hours after DOX administration. Data are shown as means ± SEM. Comparisons between multiple groups were performed using one-way ANOVA followed by a post hoc Bonferroni comparison analysis. ^∗^*P* < 0.05 compared with control. ^#^*P* < 0.05 compared with DOX alone.

We then examined NF-*κ*B activity in DOX-treated cardiomyocytes. The DNA binding activity of NF-*κ*B p65 was increased in DOX-treated cardiomyocytes. However, p65-DNA binding activity was suppressed by jaceosidin in a dose-dependent manner (Figure 2(a)). The gene expression levels of TNF-*α* and IL-6 were markedly elevated after DOX treatment. However, these increases in TNF-*α* and IL-6 were largely suppressed by jaceosidin (Figures 2(b) and 2(c)). DOX treatment impaired cardiomyocyte viability, and jaceosidin dose-dependently improved cell viability in response to DOX (Figure 2(d)). Jaceosidin treatment also dose-dependently decreased the release of CK and LDH in DOX-treated cardiomyocytes (Figures 2(e) and 2(f)).

### 3.3. Jaceosidin Treatment Attenuated DOX-Related Cardiac Injury in Mice

**Figure 3:**
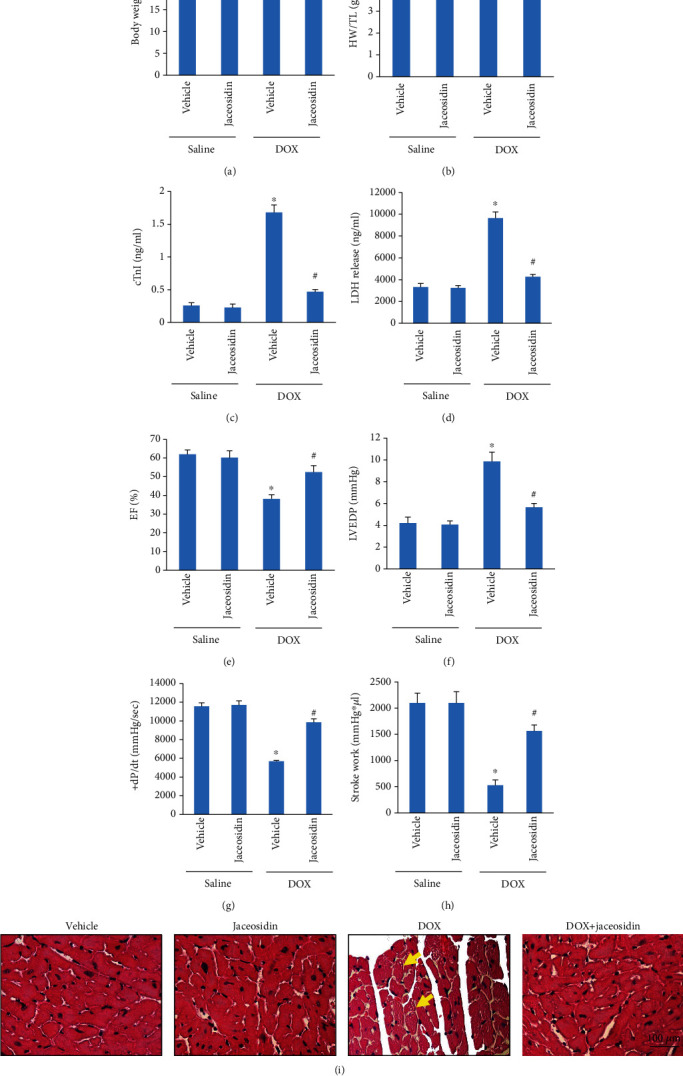
Jaceosidin (4 mg/kg) inhibited doxorubicin- (DOX-) related cardiac injury in vivo. (a) Body weight (*n* = 12). (b) The ratio of heart weight (HW) to tibia length (TL) (*n* = 12). (c, d) The plasma levels of cardiac troponin I (cTnI) and lactate dehydrogenase (LDH) (*n* = 12). (e) Ejection fraction (EF) in the mice (*n* = 8). (f, g) Left ventricular end-diastolic pressure (LVEDP) and the alteration in +dP/dt (*n* = 8). (h) The alteration in stroke work (*n* = 8). (i) Cardiomyocytes vacuolization were evaluated by HE staining. Mice were intraperitoneally injected with a single dose of DOX (15 mg/kg) to establish the acute cardiac injury model. Five days after DOX injection, blood samples and heart tissues were collected to assess cardiac injury, as reflected by (a–h). Data are shown as means ± SEM. Comparisons between multiple groups were performed using one-way ANOVA followed by a post hoc Bonferroni comparison analysis. ^∗^*P* < 0.05 compared with saline. ^#^*P* < 0.05 compared with DOX alone.

To further determine the effects of jaceosidin, mice were administered a single injection of DOX to mimic DOX-induced acute cardiac injury. As shown in Figures 3(a) and 3(b), DOX significantly decreased the body weight and the ratio of heart weight to tibal length, and these effects were largely restored by jaceosidin treatment. Furthermore, treatment with jaceosidin reduced DOX-related cardiac injury, as indicated by the decrease in cTnI and LDH release (Figures 3(c) and 3(d)). Treatment with jaceosidin also improved cardiac function in DOX-treated mice, as indicated by the improvements in EF, maximum first derivative of ventricular pressure with respect to time (+dP/dt) and stroke work, as well as the decrease in left ventricular end-diastolic pressure (LVEDP) (Figures 3(e)–3(h)). Histological examination showed that the number of cardiomyocyte vacuoles was increased in DOX-treated mice, and the change was significantly ameliorated in the DOX+jaceosidin group (Figure 3(i)).

### 3.4. Jaceosidin Treatment Inhibited Heart Oxidative Damage in DOX-Treated Mice

**Figure 4:**
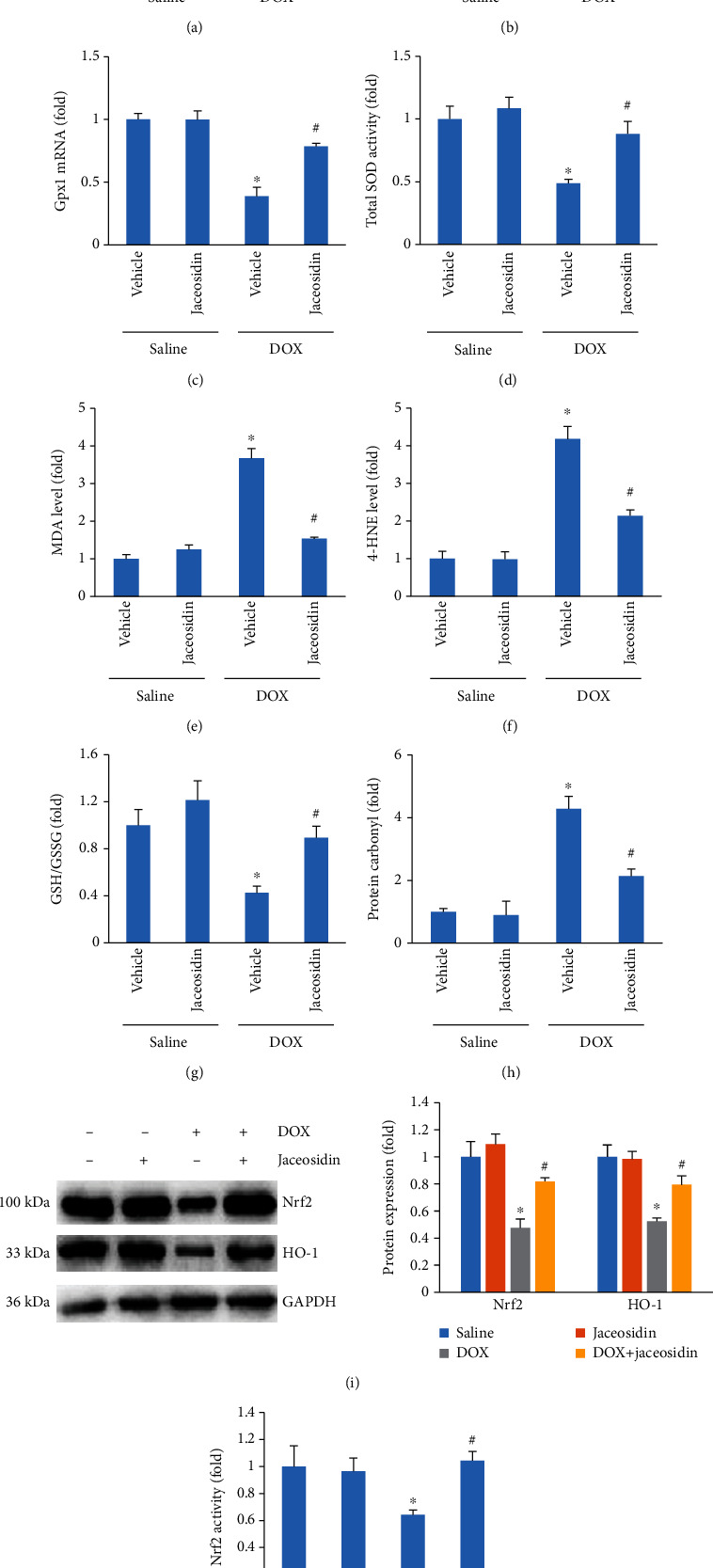
Jaceosidin (4 mg/kg) reduced oxidative stress in doxorubicin- (DOX-) treated mice. (a–c) The mRNA levels of superoxide dismutase 1 (SOD1), SOD2, and glutathione peroxidase 1 (Gpx1) in the hearts (*n* = 6). (b) Total SOD activity in the hearts (*n* = 6). (e, f) Malondialdehyde (MDA) and 4-hydroxynonenal (4-HNE) levels in the hearts (*n* = 6). (g) The ratio of glutathione (GSH) to oxidized glutathione (GSSG) in jaceosidin-treated mice (*n* = 6). (h) Protein carbonyl content in jaceosidin-treated mice (*n* = 6). (i) The protein expression of nuclear factor E2-related factor 2 (Nrf2) and haem oxygenase-1 (HO-1) in jaceosidin-treated mice (*n* = 6). (j) Nrf2 activity (*n* = 6). Mice were intraperitoneally injected with a single dose of DOX (15 mg/kg) to establish the acute cardiac injury model. Five days after DOX injection, heart tissues were collected to assess myocardial oxidative damage, as reflected by (a–j). Data are shown as means ± SEM. Comparisons between multiple groups were performed using one-way ANOVA followed by a post hoc Bonferroni comparison analysis. ^∗^*P* < 0.05 compared with saline. ^#^*P* < 0.05 compared with DOX alone.

Accumulating evidence suggests that oxidative damage plays an important role in the development of DOX-related cardiac injury [3, 20]. Our findings suggested that the reduction in the mRNA levels of SOD1, SOD2, and glutathione peroxidase 1 (Gpx1) was prevented by jaceosidin treatment (Figures 4(a)–4(c)). DOX also impaired total SOD activity, and this effect was prevented by jaceosidin treatment (Figure 4(d)). Jaceosidin reduced the increased levels of MDA and 4-HNE observed in the myocardium of vehicle-treated mice at 5 days post-DOX injection (Figures 4(e) and 4(f)). To further evaluate the oxidative stress caused by DOX, we assessed the GSH/GSSG ratio and the protein carbonyl content. DOX decreased the GSH/GSSG ratio but increased the protein carbonyl content, and these pathological alterations were prevented by jaceosidin treatment (Figures 4(g) and 4(h)). The decreased protein expression of Nrf2 and HO-1 induced by DOX was prevented by jaceosidin treatment (Figure 4(h)). Jaceosidin treatment also restored Nrf2 activity to normal levels in the hearts of DOX-treated mice (Figure 4(i)).

### 3.5. Jaceosidin Treatment Blunted the Inflammatory Response and Apoptotic Cell Death in DOX-Treated Mice

**Figure 5:**
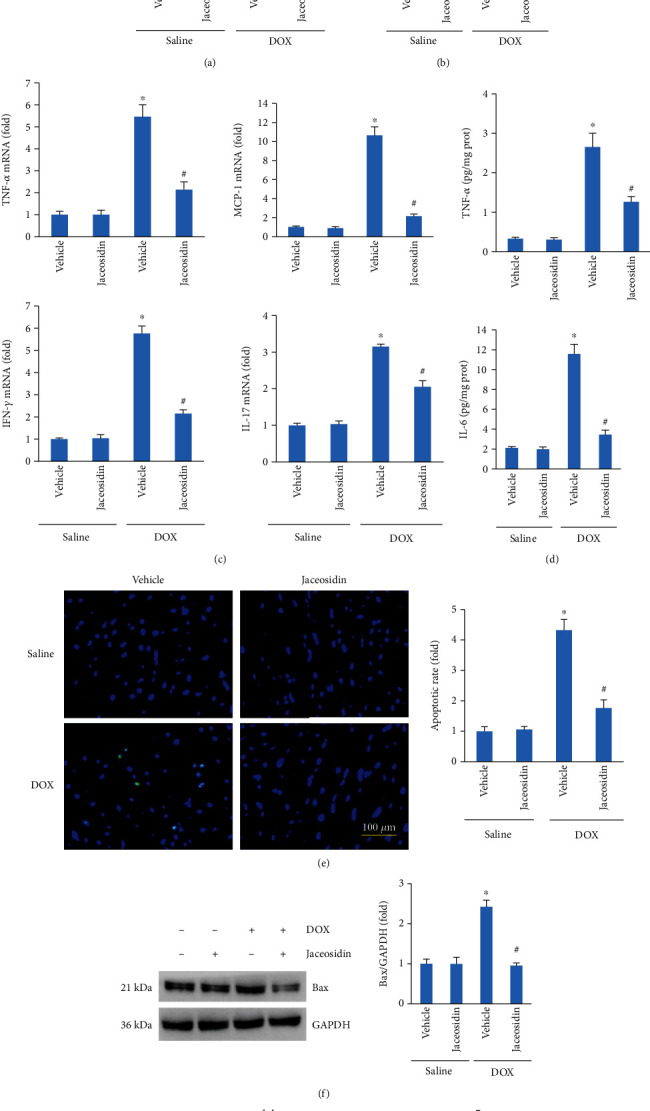
Jaceosidin (4 mg/kg) suppressed inflammation and cardiac apoptosis in doxorubicin- (DOX-) treated mice. (a, b) P65 and I*κ*B kinase *β* (IKK*β*) expression (*n* = 6). (c) mRNA levels of inflammatory factors in the hearts (*n* = 6). (d) Cytokine levels in the hearts (*n* = 6). (e) TUNEL staining in DOX-treated mice (*n* = 6). (f) Bax and Bcl-2 protein expression in DOX-treated mice (*n* = 6). (g) Caspase-3 activity in the hearts (*n* = 6). Mice were intraperitoneally injected with a single dose of DOX (15 mg/kg) to establish the acute cardiac injury model. Five days after DOX injection, heart tissues were collected to assess the myocardial inflammatory response and apoptosis, as reflected in (a–g). Data are shown as means ± SEM. Comparisons between multiple groups were performed using one-way ANOVA followed by a post hoc Bonferroni comparison analysis. ^∗^*P* < 0.05 compared with saline. ^#^*P* < 0.05 compared with DOX alone.

To determine whether jaceosidin could suppress the inflammatory response in the hearts of DOX-treated mice, we first examined NF-*κ*B p65 expression in the hearts. Jaceosidin was found to attenuate the increased expression of nuclear NF-*κ*B p65 in response to DOX (Figure 5(a)). Jaceosidin treatment also suppressed IKK*β* phosphorylation after DOX injection (Figure 5(b)). Jaceosidin treatment significantly decreased the mRNA levels of TNF-*α*, MCP-1, interferon- (IFN-) *γ*, and IL-17 in mice exposed to DOX (Figure 5(c)). The protein levels of TNF-*α* and IL-6, as detected by ELISA, were markedly elevated after DOX treatment; however, these increases were suppressed in jaceosidin-treated mice (Figure 5(d)). The TUNEL staining data revealed significant myocardial apoptosis in the DOX group compared with the saline control, and jaceosidin treatment significantly attenuated DOX-induced myocardial apoptosis (Figure 5(e)). Jaceosidin decreased Bax protein expression but increased Bcl-2 protein expression in the heart tissue of DOX-treated mice (Figures 5(f) and 5(g)). Caspase-3 activity was markedly increased in DOX-treated mice. However, the activation of caspase-3 by DOX was abolished by jaceosidin (Figure 5(h)).

### 3.6. Jaceosidin Treatment Activated Sirt1 in the Hearts of DOX-Treated Mice

**Figure 6:**
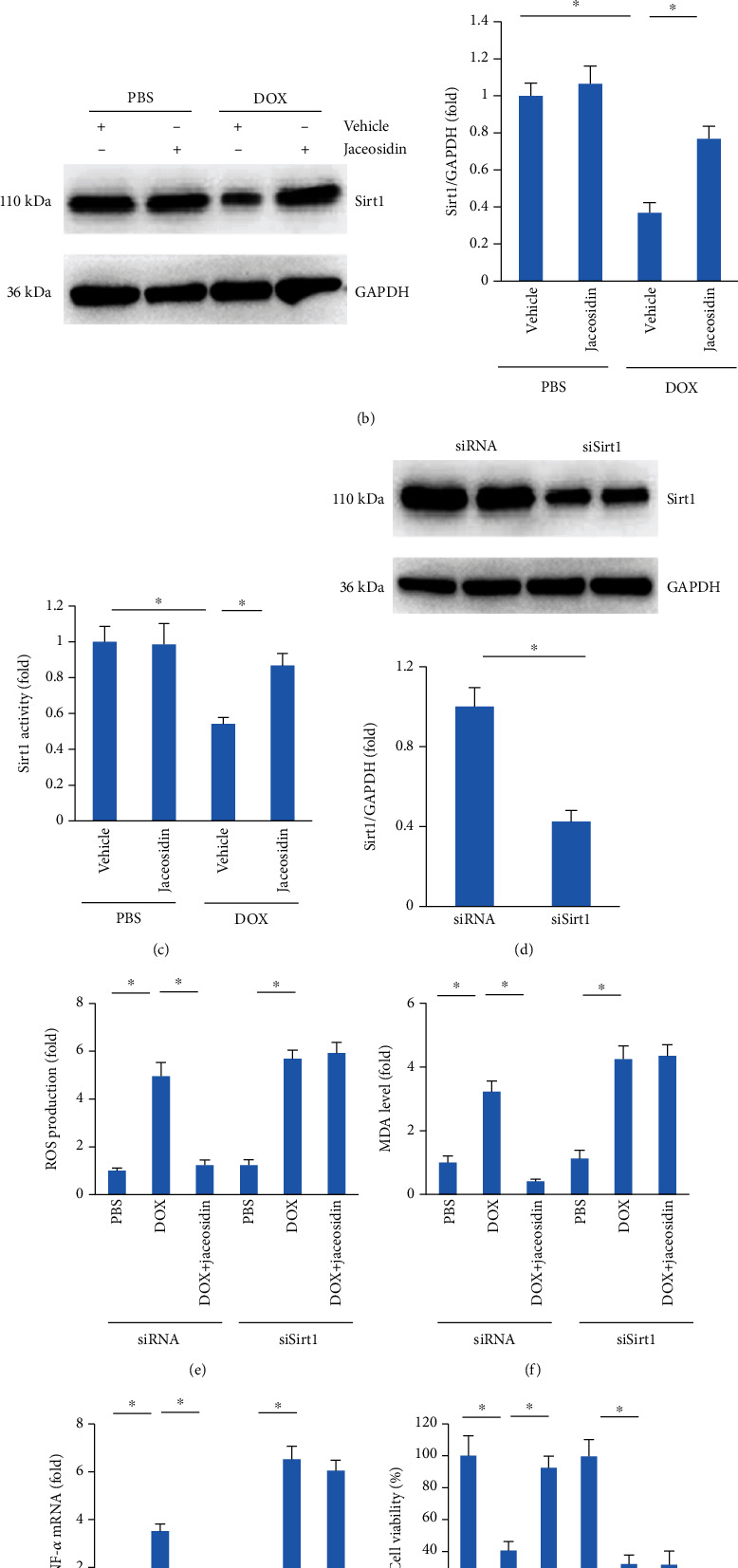
Jaceosidin (4 mg/kg) did not provide cardiac protection in sirtuin1- (Sirt1-) deficient cells. (a) Sirt1 protein expression in DOX-treated mice (*n* = 6). (b) Sirt1 protein expression in DOX-treated cells (*n* = 6). (c) Sirt1 activity in DOX-treated cells (*n* = 6). (d) Sirt1 protein expression in DOX-treated cells (*n* = 6). (e, f) ROS production and MDA content in DOX-treated cells (*n* = 6). (g) TNF-*α* mRNA level in DOX-treated cells (*n* = 6). (h) Cell viability after Sirt1 deficiency in DOX-treated cells (*n* = 6). For (b, c) and (e–h), cells were pretreated with jaceosidin (15 *μ*mol/l) 6 hours before DOX (1 *μ*mol/L) administration. To knock down Sirt1 in cardiomyocytes, NRCMs were preincubated with siSirt1 (50 nmol/l) or siRNA (50 nmol/l) for 24 hours. Data are shown as means ± SEM. For (d), comparisons were performed using two-tailed Student's *t* tests. For others, comparisons were performed using one-way ANOVA followed by a post hoc Bonferroni comparison analysis. ^∗^*P* < 0.05 versus the matched control.

We next detected the effect of jaceosidin on Sirt1 expression. As shown in Figure 6(a) and Figure S1, DOX decreased myocardial Sirt1 expression, and this effect was prevented by jaceosidin treatment. The in vitro analysis also revealed that jaceosidin increased Sirt1 expression in DOX-treated cardiomyocytes (Figure 6(b), Figure S1). The impaired Sirt1 activity in DOX-exposed cardiomyocytes was also improved after jaceosidin treatment (Figure 6(c)). To identify whether Sirt1 activation was responsible for the protective role of jaceosidin in DOX-related injury, we depleted Sirt1 in cardiomyocytes. The results showed that jaceosidin was unable to protect against ROS production, increased MDA and TNF-*α* mRNA levels, and cell loss in Sirt1-deficient cells (Figures 6(d)–6(h)).

### 3.7. Jaceosidin Had No Protective Effect in Sirt1-Inhibited Mice

**Figure 7:**
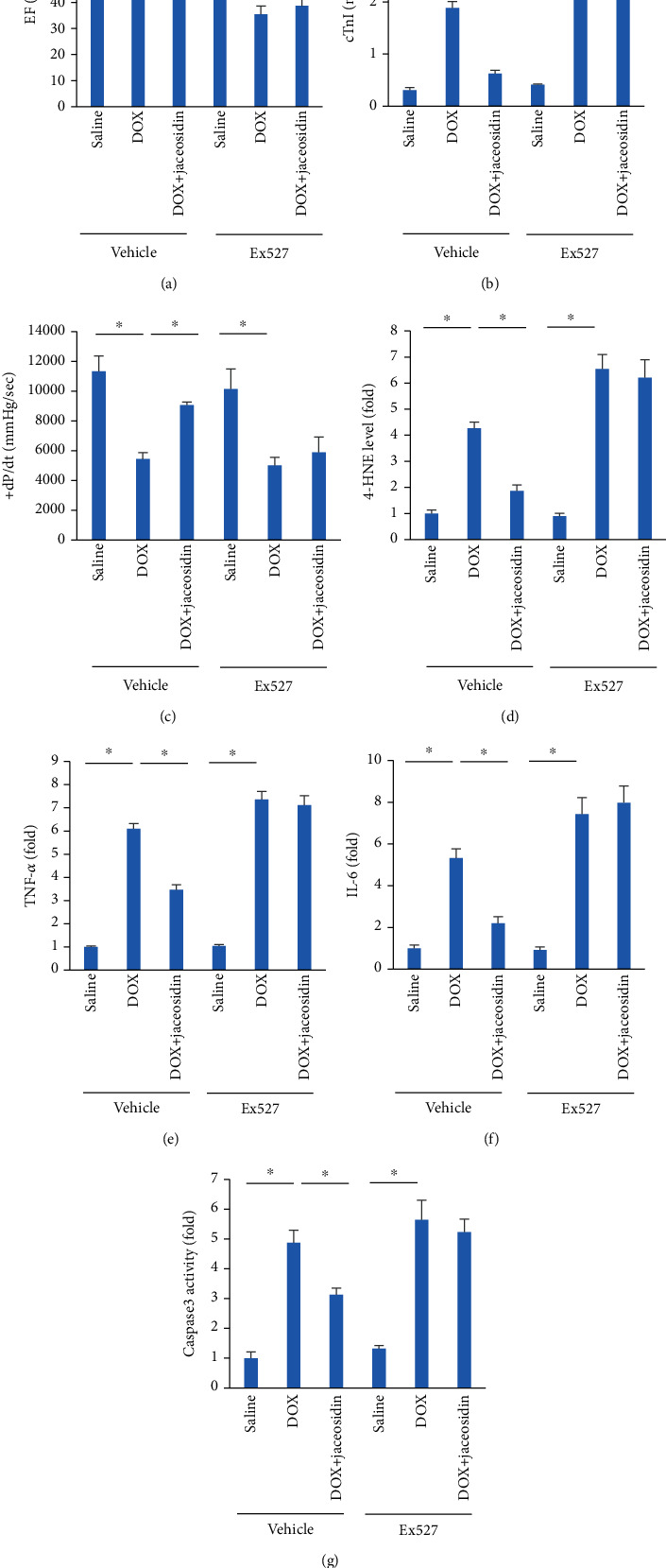
Jaceosidin (4 mg/kg) did not provide cardiac protection in mice with sirtuin 1 (Sirt1) inhibition. (a) Ejection fraction (EF) in DOX-treated mice (*n* = 8 − 10). (b) The level of cardiac troponin I (cTnI) in DOX-treated mice (*n* = 6). (C) +dP/dt in DOX-treated mice (*n* = 6). (d) The levels of 4-hydroxynonenal (4-HNE) in DOX-treated mice (*n* = 6). (e, f) The levels of tumour necrosis factor- (TNF-) *α* and interleukin- (IL-) 6 in DOX-treated mice (*n* = 6). (g) The activity of caspase-3 in mice (*n* = 6). To inhibit Sirt1 in vivo, mice were subjected to a specific inhibitor of Sirt1 (Ex527, 1 mg/kg) every other day for a total of 8 days beginning 3 days before DOX injection. Data are shown as means ± SEM. Comparisons were performed using one-way ANOVA followed by a post hoc Bonferroni comparison analysis. ^∗^*P* < 0.05 versus the matched control.

To confirm the role of Sirt1 in the effects of jaceosidin, mice were exposed to a Sirt1 inhibitor (Ex527). Extensive examinations indicated that unlike the ameliorated DOX-related cardiac injury in jaceosidin-treated mice, the extent of cardiac injury in the mice with DOX+jaceosidin+Ex527 was similar to that in mice with DOX+Ex527, as evidenced by alterations in the levels of EF, cTnI, +dP/dt, 4-HNE, inflammatory factors, and caspase-3 activity (Figures 7(a)–7(g)).

### 3.8. Oral Treatment of Jaceosidin Had No Liver or Renal Toxicity

To evaluate the potential oral toxicity of jaceosidin, mice were orally administered jaceosidin (4 mg/kg) daily for 7 days. After that, ALT, AST, and creatinine were detected. As shown in Figure S2A-C, jaceosidin administration cannot affect serum ALT, AST, and creatinine levels.

## 4. Discussion

Several ROS scavengers have been evaluated for their ability to limit DOX-induced cardiotoxicity, but with little success [21]. A low oxidant scavenging efficacy and/or the scavengers undergoing secondary reactions with other biomolecules might explain their lack of benefit [22]. Therefore, a strategy for preventing DOX-induced cardiotoxicity would help improve the quality of life of cancer patients. The present study demonstrated for the first time that jaceosidin can protect against DOX-related cardiac injury in mice and cardiomyocytes. We also found that jaceosidin supplementation can improve cardiac function and suppress DOX-induced oxidative stress, the inflammatory response, and myocardial apoptosis in mice through the activation of Sirt1. These data suggest that jaceosidin supplementation may be a promising avenue for improving cardiomyocyte survival in DOX-treated mice.

Several natural products have demonstrated the ability to protect against DOX-induced cardiotoxicity [20, 23]. However, these agents have adverse side effects, including weight loss and potentiation of doxorubicin-induced myelosuppression [24]. Jaceosidin has been shown to provide protection against several diseases [13, 14]. Jaceosidin attenuated osteoarthritic cartilage damage by blocking I*κ*B degradation in mice [25], and it inhibited contact hypersensitivity in mice by downregulating IFN-*γ* signaling in T cells [26]. Jaceosidin has not been further investigated for its biological effect in the heart, particularly for the treatment of DOX-induced cardiotoxicity. As expected, we found that jaceosidin largely reduced DOX-related cardiac injury and improved cardiac function. To the best of our knowledge, this is the first report of the protective effect of jaceosidin in DOX-induced cardiotoxicity. Considering the translational potential of the present findings, it was important to confirm that jaceosidin treatment did not compromise the oncological efficacy of DOX. We did not evaluate the effects of jaceosidin on tumour growth and metastasis. However, several lines of evidence have demonstrated that jaceosidin can suppress the growth of several tumour types [27, 28], suggesting that jaceosidin would not compromise the oncological efficacy of DOX.

DOX has a high affinity for heart tissue and can abundantly accumulate in cardiomyocytes. DOX interferes with the normal electron transport chain to increase the production of free radicals and stimulate the oxidation of membrane lipids, leading to the accumulation of the highly reactive electrophile 4-HNE, which modifies various protein functions and impacts cardiac function [29]. A previous study indicated that amelioration of oxidative damage by FNDC5 overexpression helped alleviate DOX-related cardiac dysfunction [2]. In agreement with these previous reports, we found that DOX-induced ROS production, lipid peroxidation, and the impairment of SOD activity were blunted by jaceosidin treatment. Our findings are in agreement with a previous study showing that jaceosidin eliminated free radicals in LPS-induced RAW 264.7 macrophages [30]. However, there have been conflicting reports regarding the effects of jaceosidin. Kim et al. found that jaceosidin induced apoptosis in ras-transformed human breast epithelial cells through the generation of ROS. These discrepancies might be explained by the different roles of jaceosidin in different cells. A previous study reported that DOX-induced NF-*κ*B activation occurred very early in the heart [31]. However, this activation could not be detected at 5 days after DOX injection [32]. Inconsistent with this study, we found that even at 5 days after DOX injection, NF-*κ*B activation could still be detected in the heart tissue. Further assessment revealed that the mRNA levels of inflammatory factors and cytokine production were increased in the hearts of DOX-treated mice. These increases were largely blunted in jaceosidin-treated mice, which is in agreement with a study that found that jaceosidin attenuated lung histopathological changes, inhibited the expression of NF-*κ*B, and decreased the levels of complement 3 (C3) [14]. These data imply that attenuation of oxidative damage and the inflammatory response are involved in jaceosidin-mediated inhibitory effects in DOX-related cardiac injury in mice.

Nrf2 has been recognized as one of the major cellular defence mechanisms against oxidative stress [33]. In response to ROS, Nrf2 is activated and mediates the induction of several cytoprotective enzymes [34]. It has been reported that elevated Nrf2 activity provides protection against DOX-induced cardiomyopathy [35]. Here, we tested the hypothesis that substantial Nrf2 activation, through jaceosidin induction, is involved in cardioprotection during DOX therapy. The results showed that jaceosidin restored Nrf2 protein expression in the hearts of DOX-treated mice. The mRNA levels of SOD1, SOD2, and Gpx, as well as SOD activity, were significantly decreased in the DOX group, and jaceosidin treatment was found to significantly, but not completely, prevent DOX-induced oxidative effects. These findings were consistent with a previous study showing that jaceosidin treatment increased the expression and activity of SOD in diabetic nephropathy [13]. These data suggest that jaceosidin may stimulate Nrf2-mediated downstream antioxidants to protect against DOX-induced damage.

Notably, DOX-induced oxidative stress activated apoptotic signaling and resulted in cardiomyocyte apoptosis in isolated cardiomyocytes, which was a key component in DOX-induced cardiotoxicity [36]. DOX caused the depletion of catalase and Gpx in the heart, thus creating an environment that promoted hydroxyl radical production, resulting in cytochrome c release followed by caspase-3 activation and myocardial apoptosis [7, 37]. We also found that jaceosidin largely attenuated DOX-induced myocardial apoptosis and cardiomyocyte loss. Jaceosidin treatment also decreased Bax expression and suppressed caspase-3 activity in DOX-exposed hearts. The attenuation of cell apoptosis also contributed to jaceosidin-mediated protection.

Sirt1, a histone deacetylase, is implicated in various cellular functions [38]. Sirt1 activation was shown to inhibit cardiomyocyte apoptosis in response to pathological stimuli [39]. Zhang et al. found that the Sirt1 protein level was slightly increased in response to DOX [40]. Inconsistent with this study, we observed decreased Sirt1 expression in DOX-exposed hearts. This finding was in line with a previous study that found that DOX induced a significant decrease in Sirt1 activation and that activating Sirt1 prevented DOX-related cardiotoxicity in mice [41]. Here, we found, for the first time, that jaceosidin increased Sirt1 protein expression and activity in vivo and in vitro. Sirt1 knockdown abolished the protective effects provided by jaceosidin in DOX-induced cardiotoxicity. These data clearly indicated that jaceosidin-mediated protection against DOX-related cardiac injury was mediated by the activation of Sirt1.

In conclusion, the present study demonstrated that jaceosidin treatment inhibited myocardial oxidative and inflammatory damage and reduced apoptosis, thereby improving cardiac function after DOX treatment. Our results provide experimental evidence for the application of jaceosidin in the treatment of DOX-related cardiac injury.

## Data Availability

The data that support the findings of this study are available from the corresponding author upon reasonable request.

## Supplementary Materials

Supplementary Materials Figure S1: unedited and uncropped western blot images of Figures 6 (a) and 6 (b) and Figure 6 (d). Figure S2: the level of serum ALT, AST, and creatinine (a–c). Mice were orally administered jaceosidin (4 mg/kg) daily for 7 days (*n* = 6 for each group). Data are shown as means ± SEM. Comparisons were performed using two-tailed Student's *t*-tests.
