# Supplementary material for: Protection against Doxorubicin-Related Cardiotoxicity by Jaceosidin Involves the Sirt1 Signaling Pathway
Source: Oxid Med Cell Longev. 2021 Aug 6;2021:9984330. doi: 10.1155/2021/9984330 (PMC8371661; doi:10.1155/2021/9984330)
Supplement: Supplementary Materials — Figure S1: unedited and uncropped western blot images of Figures 6 (a) and 6 (b) and Figure 6 (d). Figure S2: the level of serum ALT, AST, and creatinine (a–c). Mice were orally administered jaceosidin (4 mg/kg) daily for 7 days (n = 6 for each group). Data are shown as means ± SEM. Comparisons were performed using two-tailed Student's t-tests. [file 9984330.f1.docx]

**Supplement**

Protection against doxorubicin-related cardiotoxicity by jaceosidin involves Sirt1 signaling pathway

Yuzhou Liu*, Liying Zhou* ,Binbin Du*, Yuan Liu, Junhui Xing, Sen Guo, Ling Li, Hongrui Chen

Department of Cardiology, The First Affiliated Hospital of Zhengzhou University, Zhengzhou, Henan 450052, P.R. China

Running title: Jaceosidin against injury

*These authors contributed equally to this work

Corresponding author:

Yuzhou Liu,

Department of Cardiology, The First Affiliated Hospital of Zhengzhou University, 1 Jianshe East Road, Zhengzhou, Henan 450052, P.R. China. E-mail: 35507370@qq.com

Hongrui Chen

Department of Cardiology, The First Affiliated Hospital of Zhengzhou University, 1 Jianshe East Road, Zhengzhou, Henan 450052, P.R. China. E-mail: [chruiga@163.com](mailto:chruiga@163.com)


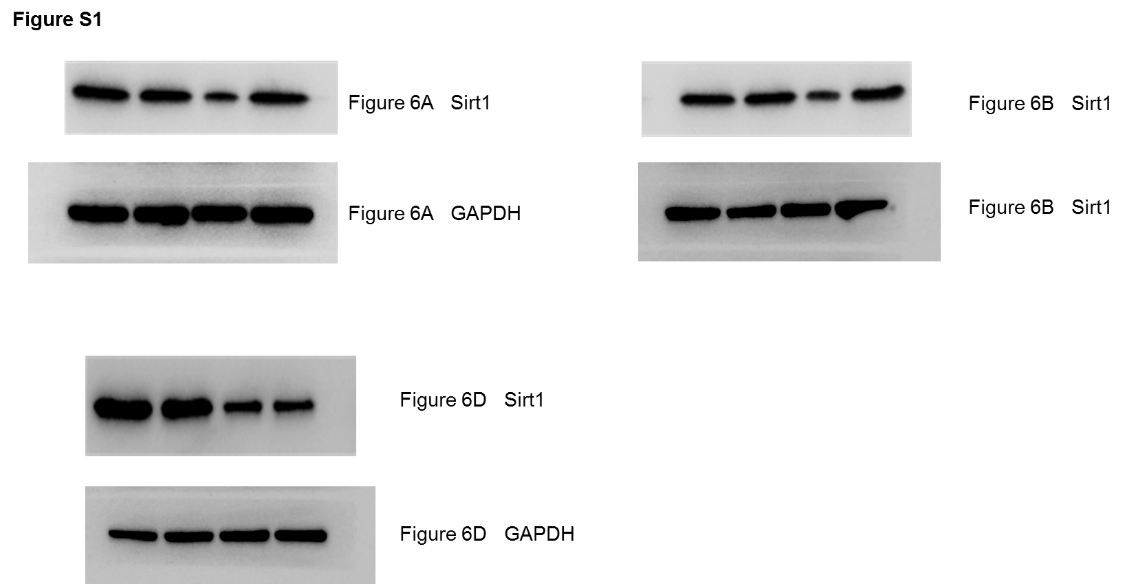


**Figure S1 Unedited and uncropped western blot images of Figure 6A-B and Figure 6D.**


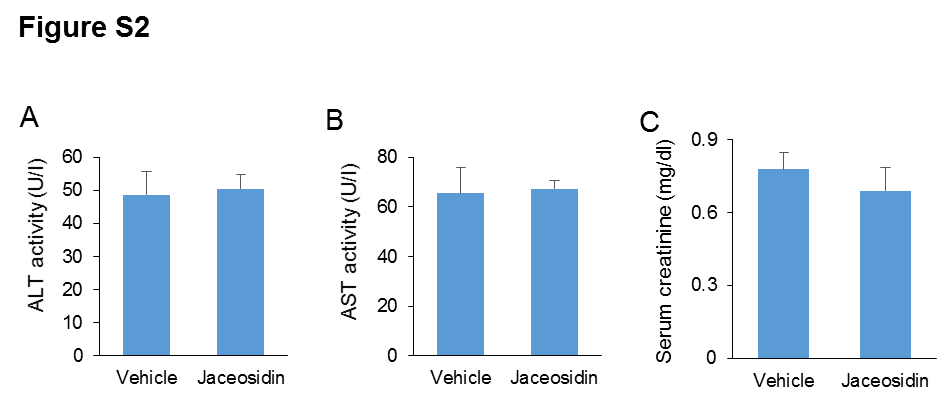


**Figure S2 The level of serum ALT, AST and creatinine (A-C). Mice were orally administered jaceosidin (4 mg/kg) daily for 7 days (n=6 for each group). Data are shown as means±SEM. Comparisons were performed using two-tailed Student's t tests.**
